# Co-morbidity and predictors of health status in older rural breast cancer survivors

**DOI:** 10.1186/2193-1801-3-102

**Published:** 2014-02-20

**Authors:** Andres Azuero, Rachel Benz, Patrick McNees, Karen Meneses

**Affiliations:** School of Nursing, University of Alabama at Birmingham, NB 1019G - 1720 2nd AVE S, Birmingham, AL 35294-1210 USA; School of Nursing, University of Alabama at Birmingham, NB 1020 - 1720 2nd AVE S, Birmingham, AL 35294-1210 USA; Schools of Nursing and Health Professions, University of Alabama at Birmingham, SHPB 630B - 1720 2nd AVE S, Birmingham, AL 35294-1212 USA; Kirchner Private Capital Group, P.O. Box 977, Gadsden, AL 35902 USA; School of Nursing, University of Alabama at Birmingham, NB 1013 - 1720 2nd AVE S, Birmingham, AL 35294-1210 USA

## Abstract

**Purpose:**

More than 66% of the 200,000 newly diagnosed annual breast cancers in the US occurs in women over 55 years. Treatment advances result in excellent survival, yet older breast cancer survivors with co-morbidity may live longer, but not better after cancer. Decline in physical function, increased social isolation, and diminished economic resources increase vulnerability among older women. Rural women represent an underserved population. The purpose is to examine associations between comorbidity and predictors of health status among older rural breast cancer survivors.

**Methods:**

Baseline data of 331 BCS age 55–90 years enrolled in the Rural Breast Cancer Survivors Study. Four surveys were used for data collection. Self-reported prescription medications were used as proxy for co-morbidity. Bivariate tests of association and multivariable recursive partitioning techniques were used for analysis.

**Results:**

Mean number of prescription medication categories reported was 3.68 (SD = 2.3; range = 0–12). Common prescription categories were: anti-hormonal, anti-hypertensive, and cholesterol- reducing agents. 69% was overweight or obese. BMI >31 was significantly associated with both poorer physical and mental health. Multivariate analyses indicated physical health status was predicted by BMI, comorbid conditions, social support, and adverse changes in economic lifestyle. The same variables, with the exception of BMI, were predictors of mental health status.

**Conclusions:**

Assessing co-morbid conditions, mental health status, social support, and economic burden after breast cancer treatment may better inform cancer survivorship care and comprehensive geriatric assessment.

## Introduction

Breast cancer is the most common female cancer with more than 200,000 new cases diagnosed each year in the United States with about 66.4% occurring in women over the age of 55 years (American Cancer Society 2013; National Cancer Institute 2013a, b). Advances in treatment have led to excellent survival and longer life expectancy (Fenlon et al. 2013). However, older women with co-morbid conditions and breast cancer represent a growing population who may be living longer, but not necessarily better after completion of successful cancer therapy (Fenlon et al. 2013; Bellury et al. 2012; Ganz et al. 2003). Decline in physical function, increased social isolation, and diminished economic resources may increase vulnerability among older breast cancer survivors (Extermann 2007; Girones et al. 2010; Sehl et al. 2013; Steptoe et al. 2013).

Cancer survivors with co-morbidities have poorer physical and mental health compared with those without cancer (Smith et al. 2008). Among older breast cancer survivors, arthritis, hypertension, diabetes, and overweight/obesity are most frequently reported (Bellury et al. 2012; Weaver et al. 2013). The greater the number of co-morbid conditions is associated with poor functional ability, increased symptoms, declining overall general health and quality of life, and increase in the number of physician visits (Bellury et al. 2012; Bellury et al. 2013; Deimling et al. 2009; Hays et al. 2013; Kurtz et al. 2005; Yoon et al. 2008). Social support mediates physical and mental health status in older adults (Lachman & Agrigoroaei 2010; Seeman 2000; White et al. 2009); and refers to real or perceived resources provided by others that enable a person to feel cared for, valued, and part of a network of communication and mutual obligation (Stroebe 2000). High levels of social support have been associated with improved health status, and reduced risk of physical and mental illness (Lachman & Agrigoroaei 2010; Seeman 2000; White et al. 2009). Social support has also been found to improve feelings of vulnerability (Seeman 2000).

Despite the availability of studies that examine co-morbidity and cancer, few focus on co-morbidity in rural cancer survivors. While the reasons are unknown, the paucity of data is consistent with the reality that rural cancer populations are typically underserved and understudied (Befort & Klemp 2011). In one of the few reported studies of survivors living in a semi-rural setting, Beck et al., explored symptom experience, health-related quality of life, and functional performance in 52 older adults with cancer, of whom 22 were semi-rural survivors (Beck et al. 2009). The investigators found that 88% of the entire sample, including semi-rural survivors with co-morbid disease, had poor physical status and greater symptom intensity (Beck et al. 2009). Given this paucity of research, and in an attempt to better understand unique challenges and needs of rural breast cancer survivors, the investigators analyzed baseline data from the Rural Breast Cancer Survivors study, a population-based sample of older rural breast cancer survivors.

The specific aims of the present study are to: (1) examine associations between co-morbid conditions, and physical and mental health status among older rural breast cancer survivors; (2) identify predictors of physical and mental health status of older rural breast cancer survivors; and (3) explore challenges to improve cancer survivorship care and comprehensive geriatric assessment.

## Materials and methods

Data were drawn from the parent study, the Rural Breast Cancer Survivors Study (RBCS), a population-based study conducted in the State of Florida evaluating the dissemination and implementation of cancer survivorship interventions among rural breast cancer survivors. The RBCS was approved by Institutional Review Boards and the State of Florida Department of Health (DOH). Participants were identified through Florida cancer registry. Eligibility criteria included: at least 21 years of age, Florida rural residence, telephone access, and diagnosis of Stage 0-III breast cancer in the previous three years. Survivors receiving anti-hormonal therapy were eligible. Survivors with metastatic disease, men with breast cancer, and those lacking reliable telephone access were not eligible.

### Rural eligibility

Rural eligibility was established using one of two criteria: (1) residence in one of 33 Florida rural counties designated by Florida statute; or (2) residence in a rural area of an urban county having an Index of Research Access (IRA) score equal to or greater than 4 (Florida Department of Health 2007; The Authors 2012). The IRA indicates the level of difficulty of treatment or research access, providing a description that cannot be obtained by county residence alone. Information and details for calculating the IRA are reported elsewhere (The Authors 2012).

### Consent process and data collection

A total of 432 rural BCS enrolled. Data were collected at baseline and every three months for a total of 12 month participation. Self-report data from participants was collected via the telephone. Data for the present study were derived using baseline data from 331 rural BCS ages 55 to 90 years.

### Variables of interest

The following variables were examined: (1) health status, (2) co-morbid conditions, (3) sociodemographic and treatment characteristics, and (4) social support.

#### ***Health status***

The Medical Outcomes Study Short Form v1 (SF-36) was used to assess self-reported health status (Ware & Sherbourne 1992). The SF-36 has 36 items that aggregate into two overall summary scores: the Physical Component Summary score (PCS) and the Mental Component Summary score (MCS). The PCS was used as measure of physical health status, and the MCS was used as a measure of mental health status. Norm scores range from 0 to 100 where higher scores indicate better functioning and health status. In the present study, the PCS and the MCS had alpha reliability of .93 and .90 respectively.

#### ***Co-morbid conditions***

The reported number of categories of prescription medication was used as a proxy for co-morbid conditions. This variable was measured using 22 items contained in the Work and Finances Inventory used in prior studies by the investigators (The Authors 2007). Three additional items related to economic events since end of cancer treatment.

#### ***Sociodemographic and treatment characteristics***

The Breast Cancer Survivor Sociodemographic Treatment Survey has been used in prior work by the investigators (The Authors 2007). The survey consists of 26 items: 12 sociodemographic; 12 on breast cancer treatment; two on weight and height.

#### ***Social support***

The Medical Outcomes Study Social Support (MOS-SSS) questionnaire assessed social support (Sherbourne & Stewart 1991). The 19 items are averaged and re-scaled into an overall functional social support index, with scores ranging from 0 to 100, with higher scores indicating more social support. Alpha reliability for the functional social support index was 0.96.

### Data analysis

Participant characteristics were tabulated, including frequencies for each prescription category. Bivariate tests of association between SF-36 PCS and MCS scores and participant characteristics were conducted using general linear models to produce ANOVA-equivalent tests. The prescription categories most strongly associated with SF-36 PCS and MCS scores were tabulated. To determine covariate-adjusted associations between number of prescriptions and the SF-36 PCS and MCS scores, multivariable analyses were conducted considering sociodemographics, cancer treatment, and social support as additional predictors of SF-36 PCS and MCS scores.

The multivariable analyses were conducted using conditional inference tree modeling, a statistical technique for recursive partitioning (Everitt & Hothorn 2009). Tree modeling allows detection of complex interactions among predictors that cannot be uncovered via traditional linear model analysis (The Authors 2011a). The conditional inference tree modeling procedure conducts predictor selection and partitioning of the predictor space via statistical tests (i.e., permutation tests, a class of non-parametric tests), while applying a strong control for multiple testing using Bonferroni adjustments to prevent over-fitting. Unlike linear models, tree models are not typically described by equations but are shown graphically.

Prior to conducting the multivariable analyses, a small proportion of missing data (approximately 1%) was imputed using the Multivariate Imputation by Chained Equations (MICE) procedure, with CART (Classification And Regression Trees) as underlying modeling technique (Hothorn et al. 2006; van Buuren & Groothuis-Oudshoorn 2011). A single imputation was used due to the minimal amount of missing data points. The above analyses were conducted using SAS v9.3 statistical software, along with the Packages Party and MICE in the R statistical software v3.0.1 (Everitt & Hothorn 2009; van Buuren & Groothuis-Oudshoorn 2011). Statistical significance was held at the traditional 0.05 level.

## Results

### Sociodemographic characteristics

Sociodemographic characteristics among the sample are listed in Table 1 (left columns 1 and 2). The sample was divided into three groups: young old (age 55–64), old (65–70), and the very old (71–90) years. Age showed a bimodal distribution with the young old and the very old having the largest number of participants (71.6%). The majority were Caucasian (95%), had technical school or some college education (34.5%), retired (58.3%), and health insurance (95.8%). About 71% was married or living with a partner. More than 65% had social support scores greater than 76% indicating good social support. Twenty-three percent reported incomes under $30,000. Nearly 39% reported a decrease in income level; 25.4% reported an adverse change in economic lifestyle; with 10.6% reported having to borrow money since completion of cancer therapy.

**Table 1:** **SocioDemographic characteristics and bivariate test of association with SF-36 Physical Component Summary Score (PCS) and Mental Component Summary Score (MCS) (N = 331)**

Characteristic	n (%)	SF-36 PCS,	SF-36 MCS,
		mean (SD)	mean (SD)
**Age groups**		**P = 0.339**	**P < .001**
55-64	125 (37.8)	45.8 (11.27)	46.35 (11.97)
65-70	94 (28.4)	44.35 (10.89)	49.63 (11.52)
71-90	112 (33.8)	43.82 (9.83)	52.21 (10.62)
Number of prescription categories*		**P < .001**	**P = 0.164**
0-2	110 (33.3)	47.49 (10.75)	50.41 (11.98)
3-4	115 (34.7)	45.39 (9.87)	49.77 (10.69)
5-12	106 (32.0)	41.13 (10.59)	47.54 (12.14)
**Marital status**		**P = 0.354**	**P = 0.404**
Never married	5 (1.5)	49.5 (5.17)	52.08 (8.34)
Married or living w/partner	235 (71)	45.03 (10.68)	49.72 (11.15)
Separated/divorced/widowed	91 (27.4)	43.65 (10.93)	47.94 (12.94)
**Ethnicity**		**P = 0.421**	**P = 0.701**
Caucasian	315 (95.2)	44.61 (10.67)	49.32 (11.62)
Non-Caucasian Minority	16 (4.8)	46.82 (11.39)	48.17 (12.29)
**Education**		**P = 0.227**	**P = 0.025**
< High school	19 (5.7)	43.08 (10.46)	44.21 (11.05)
High school graduate	75 (22.7)	44.7 (10.29)	48.61 (12.27)
Technical school/some college	114 (34.5)	43.22 (10.48)	48.03 (12.68)
Completed college	76 (22.9)	46.53 (10.87)	52.44 (9.43)
Postgraduate	47 (14.2)	46.12 (11.47)	50.27 (10.42)
**Employment status**		**P < .001**	**P = <.001**
Full-time	56 (16.9)	48.43 (8.94)	48.15 (10.06)
Part-time	48 (14.5)	46.42 (10.42)	48.69 (11.77)
Retired	193 (58.3)	44.13 (10.42)	50.99 (11.47)
Homemaker	13 (3.9)	43.72 (12.83)	44.48 (11.27)
Disability	12 (3.6)	31.71 (11.96)	35.47 (11.86)
Unemployed	9 (2.8)	43.93 (10.15)	47.86 (11.18)
**Health insurance**		**P = 0.581**	**P = 0.008**
No	14 (4.2)	43.17 (12.06)	41.25 (13.1)
Yes	317 (95.8)	44.79 (10.65)	49.62 (11.46)
BMI category*		**P < .001**	**P = 0.645**
Underweight (<18.5)	4 (1.2)	43.3 (12.17)	55.65 (3.75)
Normal (18.5 to 24.9)	98 (30.3)	49.28 (9.26)	49.12 (11.77)
Overweight (25 to 29.9)	107 (33.2)	46.21 (10.04)	49.87 (11.63)
Obese (≥30)	114 (35.3)	39.76 (10.26)	48.85 (11.35)
**MOS overall social support score**		**P = 0.018**	**P < .001**
12-50	45 (13.6)	40.55 (10.74)	40.45 (13.09)
51-75	69 (20.8)	45.7 (10.36)	46.32 (10.96)
76-100	217 (65.6)	45.28 (10.65)	52.03 (10.36)
**Family income**		**P = 0.023**	**P = 0.002**
$20,000 or less	55 (16.6)	43.61 (11.56)	45.08 (13.33)
$20,001 to $30,000	54 (16.3)	41.83 (10.06)	47.56 (13.98)
$30,001 to $40,000	27 (8.2)	43.46 (12.22)	47.47 (9.19)
$40,001 to $50,000	36 (10.9)	46.36 (9.7)	48.89 (10.56)
Greater than $50,000	110 (33.2)	47.22 (9.61)	50.62 (10.33)
Declined to answer	49 (14.8)	43.04 (11.62)	54.05 (9.58)
**Decrease in income level** ^**a**^ *		**P = 0.283**	**P = 0.023**
No	202 (61.1)	45.22 (10.63)	50.42 (10.82)
Yes	129 (38.9)	43.92 (10.8)	47.45 (12.65)
**Borrowed money** ^**a**^ *		**P = 0.066**	**P < .001**
No	296 (89.4)	45.09 (10.5)	50.17 (11.03)
Yes	35 (10.6)	41.58 (11.92)	41.69 (13.87)
**Changed economic lifestyle** ^**a**^ *		**P = 0.043**	**P < .001**
No	247 (74.6)	45.42 (10.5)	51.07 (10.65)
Yes	84 (25.4)	42.68 (11.07)	43.99 (12.8)

Notes: P-values from ANOVA tests; BMI = Body Mass Index; MOS = Medical Outcomes Study.

*Counts < 331 due to missing responses; BMI categories from Centers for Disease Control and Prevention.

^a^Since end of cancer treatment.

### Body weight

Body mass index (BMI) was divided into four categories: underweight, normal weight, overweight and obese, based on CDC definitions (Centers for Disease Control 2011). More than 33% were overweight; and more than 35% were obese.

### Treatment characteristics

Table 2 displays cancer treatment characteristics. Nearly 80% completed primary breast cancer treatment more than a year prior to entering the study. The majority had lumpectomy (60.7%) and radiation therapy for local control of disease (70.7%), and chemotherapy (52.3%) for regional and systemic control of disease. Nearly 66% received hormonal blocking agents to reduce the risk for recurrence.

**Table 2:** **Cancer treatment characteristics and bivariate test of association with SF-36 Physical Component Summary (PCS) and Mental Component Summary (MCS) Scores (N = 331)**

Characteristic	n (%)	SF-36 PCS,	SF-36 MCS,
		mean (SD)	mean (SD)
Months since end of treatment *		**P = 0.709**	**P = 0.091**
≤12	66 (20.4)	43.84 (11.04)	46.43 (12.59)
13-24	181 (56.1)	45.12 (10.43)	50 (11.49)
25+	76 (23.5)	44.71 (11.27)	49.82 (11)
**Surgery received**		**P = 0.941**	**P = 0.009**
Lumpectomy	201 (60.7)	44.81 (11.14)	50.32 (10.88)
Mastectomy	98 (29.6)	44.72 (10.01)	48.94 (11.84)
Bilateral mastectomy	32 (9.7)	44.11 (10.13)	43.59 (14.11)
**Chemotherapy**		**P = 0.851**	**P = 0.004**
No	158 (47.7)	44.83 (10.55)	51.17 (11.59)
Yes	173 (52.3)	44.61 (10.86)	47.52 (11.43)
**Radiation**		**P = 0.906**	**P = 0.063**
No	97 (29.3)	44.61 (10.16)	47.4 (13.4)
Yes	234 (70.7)	44.76 (10.93)	50.02 (10.78)
**Hormonal therapy** ^**a**^		**P = 0.496**	**P = 0.446**
No	113 (34.1)	44.16 (11.15)	48.59 (13.37)
Yes	218 (65.9)	45.01 (10.47)	49.62 (10.64)
**Treatment mix**		**P = 0.184**	**P = 0.002**
Surgery only	50 (15.1)	46.68 (9.7)	50.96 (12.71)
Surgery, chemotherapy	47 (14.2)	42.4 (10.28)	43.61 (13.21)
Surgery, radiation	108 (32.6)	44 (10.86)	51.27 (11.11)
Surgery, chemo, radiation	126 (38.1)	45.42 (10.99)	48.95 (10.41)

Notes: P-values from ANOVA tests; * Counts < 331 due to missing responses;

^a^On hormonal treatment at the time the data were collected.

### Prescriptions

Table 3 shows the mean number of 3.68 prescription categories with a range of 0–12 prescription categories reported by participants. The most common prescription category was anti-hormonal agents for cancer reported by 66.2% (n = 219) of participants. High blood pressure and high cholesterol were second and third highest with 48.6% (n = 161) and 38.7% (n = 128) respectively, indicating that cardiovascular disease was the most commonly treated co-morbidity in this sample. Four medication categories reported are commonly associated with breast cancer treatment or side effects include: pain (19.9%), depression (14.8%), insomnia/sleep (14.2%), anxiety (13.9%) and hot flashes (5.4%).

**Table 3:** **Frequencies of prescription categories reported by older rural breast cancer Survivors (n = 331)**

Medication category	n	%
Cancer (hormonal therapy)	219	66.2
High blood pressure	161	48.6
High cholesterol	128	38.7
Thyroid	66	20.0
Pain	66	19.9
Heartburn	56	16.9
Depression	49	14.8
Insomnia/sleep	47	14.2
Anxiety	46	13.9
Osteoporosis	43	12.9
Diabetes	40	12.1
Allergies	37	11.2
Edema/swelling	28	8.5
Asthma	21	6.3
Bladder spasms	18	5.4
Hot flashes	18	5.4
Infections	11	3.3
Angina	9	2.7
Ulcers	5	1.5
Congestive heart failure	4	1.2
Cough	3	0.9
Other 1	96	29
Other 2	35	10.6
Other 3	13	3.9
**Prescription category counts per participant**		
Mean (SD)	3.68 (2.3)	
Median	3	
Range	0 -12	

The larger the number of prescription categories was significantly associated with lower PCS score (Table 1). A similar relationship was observed between the number of prescriptions and the MCS score, but did not reach statistical significance. Individual prescription categories most strongly associated with PCS scores were pain, heartburn, diabetes, bladder spasms, infections, allergies, and other-not listed (Table 4)**.** Medication categories most strongly associated with MCS scores were anxiety, depression, insomnia, and diabetes (Table 5). Taking medication for diabetes was associated with both lower PCS and MCS scores.

**Table 4:** **Reported prescription categories associated with SF-36 Physical Component Summary (PCS) Score (n = 331)**

Prescription	Reported?				P*
	No		Yes		
	n	mean (SD)^a^	n	mean (SD)^a^	
a) Pain	265	46 (10.1)	66	39.5 (11.4)	<.001
b) Heartburn	275	45.7 (10.4)	56	39.8 (10.8)	<.001
c) Diabetes	291	45.4 (10.6)	40	39.5 (9.8)	<.001
d) Bladder spasms	313	45.2 (10.6)	18	36.9 (10.5)	0.002
e) Infections	320	45 (10.6)	11	36.9 (10.8)	0.014
f) Allergies	294	45.2 (10.6)	37	40.9 (10.9)	0.019
e) Others not listed	235	45.6 (10.4)	96	42.5 (11.2)	0.014

^a^SF-36 PCS score; *t-test.

**Table 5:** **Reported prescription categories associated with SF-36 Mental Component Summary (MCS) Score (n = 331)**

Prescription	Reported?				P*
	No		Yes		
	N	mean (SD)^a^	n	mean (SD)^a^	
a) Anxiety	285	50.4 (11.1)	46	42.4 (12.8)	<.001
b) Depression	282	50.3 (11.3)	49	43.1 (11.7)	<.001
c) Insomnia/sleep	284	50.2 (11.2)	47	43.9 (13)	<.001
d) Diabetes	291	50.1 (11.2)	40	43.5 (13.4)	<.001

^a^SF-36 MCS score; *t-test.

### Participant characteristics

Individual characteristics significantly associated with PCS scores (Table 1, **column 3 &4**) included employment status, BMI, social support, family income, and changes in economic lifestyle. Characteristics significantly associated with MCS scores (Tables 1 and 2) were age, education level, employment status, health insurance status, social support, family income, economic events since end of cancer (i.e., decrease in income, borrowing money, and adverse changes in economic lifestyle), type of surgery, and chemotherapy.

**Figure 1:**
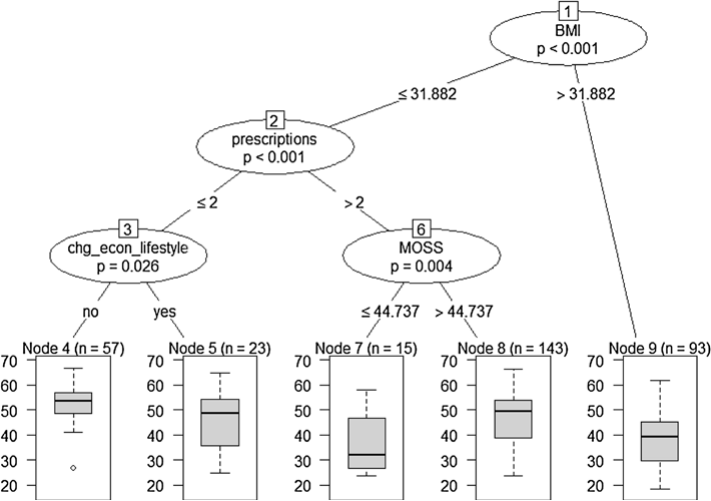
**Conditional inference tree model for SF-36 Physical Component Summary (PCS) score (n=331).** Notes: Correlation between actual and predicted= 0.47, P<.0001; Predicted SF-36 PCS per terminal node: Node 4= 52.54; Node 5= 45.72; Node 7= 36.70; Node 8= 46.47; Node 9= 38.46.

The non-linear multivariable tree model for PCS scores (Figure 1) showed that the combination of factors including: BMI, number of prescription categories, adverse changes in economic lifestyle, and social support were strong predictors of PCS scores. Among participants with BMI less than 31.8, the 15 participants having the lowest average PCS of 36.7 (Figure 1, **terminal node 7**), reported more than two prescription categories in combination with low levels of social support. In contrast, the 57 participants with the highest average PCS of 52.5 (Figure 1, **terminal node 4**) reported no more than two prescription categories, and no adverse changes in economic lifestyle since end of cancer treatment. Ninety-three participants with high BMI (over 31.88) reported low average PCS of 38.5 (Figure 1, **terminal node 9**), or approximately one standard deviation below the U.S. adult average.

**Figure 2:**
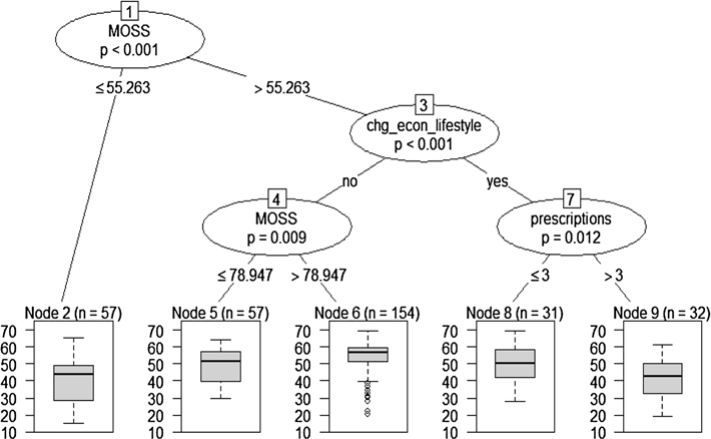
**Conditional inference tree model for SF-36 Mental Component Summary (MCS) score (n=331).** Notes: Correlation between actual and predicted= 0.48, P<.0001; Predicted SF-36 MCS per terminal node: Node 2= 40.18; Node 5= 49.06; Node 6= 54.21; Node 8= 50.43; Node 9= 41.53.

The non-linear multivariable tree model for MCS scores (Figure 2) showed that the combination of social support, adverse changes in economic lifestyle, and the number of prescription categories were strong predictors of MCS scores. The tree model revealed that the number of prescription categories was an important factor among 63 participants having at least midrange levels of social support, and who also reported adverse changes in economic lifestyle since end of cancer treatment. Among these participants, 31 who reported no more than three prescription categories (Figure 2, **terminal node 8**) had an average MCS of 50.4 which was almost a standard deviation higher than the average among 32 who reported more than three prescription categories estimated at 41.5 (Figure 2, **terminal node 9**). The 57 participants with less than midrange social support scores (Figure 2, **terminal node 2**) had the lowest average MCS at 40.2. In contrast, 154 participants who reported no adverse changes in economic lifestyle and levels of social support of at least 79% of the maximum scores had the highest average MCS of 54.2, or 40% of a standard deviation above the general U.S. adult average MCS score.

## Discussion

Significant associations between co-morbidity and health status were noted. The majority was overweight or obese which was significantly associated with both poor physical health and poor mental health. Implications of this finding are exacerbated by the reality that increased BMI is a risk factor for many diseases including cardiovascular disease, selected cancers, diabetes, and other ailments (World Health Organization 2013).

The second comorbidity after cancer was cardiovascular disease. Anti-hypertensives and cholesterol-reducing medications were second and third in frequency. While these percentages are lower than the national average for these conditions, they represent an important health concern among aging women with breast cancer (American Heart Association 2013a, b).

Over 12% of survivors reported medication for diabetes. In a sample of survivors of mixed cancers, Stava et al. found that diabetes affected health and psychosocial well-being outcomes (Stava et al. 2007). Our findings showed similar results. Furthermore, findings showed that diabetes had a significant association with both poor physical and mental health status, indicating a compounding effect of this particular disease on one's overall health and function. The percentage reporting medication use for diabetes is slightly smaller than the national incidence (16.75%) of women older than 55 years (Centers for Disease Control and Prevention 2011). Lower reported usage of diabetes medication compared to the national incidence raises the possibility of greater risk due to undiagnosed, and thus, unmanaged diabetes.

Survivors continued to experience late effects of cancer beyond two years after treatment. Problems with pain, depression, insomnia/sleep problems, anxiety, and hot flashes are bothersome late effects of treatment. These symptoms can disrupt activities of daily living and influence overall perceptions of quality of life.

### Predictors of health status

#### ***Physical health status***

The tree model illustrating significant predictors of physical health status was the combination of several factors including BMI, number of prescription categories, adverse changes in economic lifestyle, and social support. Older rural breast cancer survivors who were obese reported, on average, poor physical health. However, older rural breast cancer survivors who were not obese but have more than two co-morbid conditions and low levels of social support reported, on average, the poorest physical health. In contrast, older rural breast cancer survivors who were not obese and reported having less than two co-morbid conditions with no adverse changes in economic lifestyle reported the highest average physical health. Overall, participants having more than two comorbid conditions or were obese had poor levels of physical health.

#### ***Mental health status***

The tree model also illustrated that significant predictors of mental health status were the combination of co-morbid conditions, social support, and adverse economic changes. The tree model revealed that the number of comorbid conditions was a significant predictor of mental health among participants having at least moderate levels of social support and who experienced adverse economic events since completion of cancer treatment. Those with three or less co-morbid conditions had significantly higher mental health compared with those having more than three. Those with high levels of social support and no adverse economic lifestyle events had the highest level of mental health. Those with less than moderate levels of social support had the lowest level of mental health.

### Challenges in survivorship care and geriatric assessment

This sample of older rural breast cancer survivors reflected general aspects of the rural population of women living in the State of Florida. About 16% reported annual household incomes of less than $20,000 which is lower than the state average poverty level of 17.1% (Spotlight on Poverty and Opportunity 2013). More than 94% reported having health insurance, a figure greater than the state average of 80%, and received standard treatment consistent with current breast cancer treatment guidelines (Spotlight on Poverty and Opportunity 2013; National Comprehensive Cancer Network 2013).

Participants reported a decrease in income level, changes in economic lifestyle, and having to borrow money after breast cancer therapy ended. Limited resources, economic burden, and unexpected economic events can have an impact on health. Previous data indicates that women with metastatic cancer and older breast cancer survivors receiving chemotherapy experience economic burden (Sail et al. 2012; Wan et al. 2013). Our data showed that economic burden also occurred in older women with early stage I and II disease. Rural breast cancer survivors may carry disproportionate economic burden because they may have larger travel expenditures and out of pocket costs to seek cancer follow-up care and survivorship services (The Authors 2011b). This burden may be exacerbated by less disposable income or savings available.

Social and relational support is critical to one’s life, and its protective role in health maintenance and quality of life are well known (Steptoe et al. 2013; Barber 2013; Hanratty et al. 2013). Our findings further support the vital function of social support, and underscore the need to assess available social support that may buffer decline among older rural breast cancer survivors. In addition, broader socio-environmental factors such as rurality, greater than three comorbid conditions, and the experience with adverse economic events may be particularly critical to assess in breast cancer survivors.

The combination and influence of co-morbidity, social support, and adverse economic lifestyle events on physical and mental health underscore the importance of comprehensive and multi-dimensional evaluation beyond standard cancer survivorship care planning. Comprehensive geriatric assessment can be incorporated with cancer survivorship care planning for earlier identification of vulnerable elders to maintain independence, and plan for necessary resources (Balducci 2003; Bellury et al. 2011). Primary care providers who are involved in cancer care of older cancer survivors remain a critical group to improve current models of survivorship care (Bowman et al. 2010).

While all breast cancer survivors may be at risk of not receiving services that may make a difference in their post-treatment quality of life, rural dwelling women may be at particular risk. Understanding the exact nature of similarities and differences in rural versus urban dwelling women may help ameliorate some of the these risks and provide guidance as to precise nature of services that are warranted.

### Future research implications

Findings documented the differential types of co-morbidity among older rural breast cancer survivors, and the potential implications for declining physical and mental health. Development of future interventions designed to promote better physical and emotional well-being, social support, and communication among older rural breast cancer survivors are warranted.

Methodological considerations serve to caution researchers against excluding older cancer survivors with co-morbidity from study participation (McCaskill-Stevens & Abrams 2011). Given the dearth of information concerning older, rural breast cancer survivors in the United States, further attention can be paid to include this population in cancer research. However, the increasing complexity in this patient population may not be fully addressed by current cancer research paradigms (Stommel & Schoenborn 2009). Additional work and understanding are needed.

### Limitations

Several limitations are noted. First, participants with either an undiagnosed or untreated health condition would lead to under-detection in the data. Second, while the total number of co-morbid conditions listed in the survey instrument was large (n = 22), the list of co-morbid conditions was not comprehensive, and therefore certain health conditions may not have been detected. For example, some participants described having joint or musculoskeletal pain. This type of pain is a known side effect of aromatase inhibitor therapy for which they may have been taking pain medication. Yet, a specific prescription category for joint or musculoskeletal pain and arthritis was not contained in the study instrument. Third, BMI was calculated by self-report of weight and height, which may have resulted in inaccurate estimates of actual BMI, particularly at the very low or very high end of weight (McCaskill-Stevens & Abrams 2011).

Finally, the possible critically important role of diabetes in this population is recognized. Yet, we do not know if the present sample had a lower incidence of diabetes or if they had a higher incidence of undiagnosed diabetes. If the latter is true, this would place older rural breast cancer survivors at an even greater risk. Thus, it is an important issue to address in future research.

## Conclusion

The intersect among three factors: aging, cancer, and co-morbid conditions represent characteristics of a growing population of cancer survivors having unique survivorship needs that may place them at a higher risk of vulnerability, economic changes, and decline in social support after treatment ends. The implications and need for future research into the quality of life of older breast cancer survivors are broad as the population continues to age and people live longer after diagnosis. The complexity of needs experienced by aging breast cancer survivors need to be addressed. Because of the multifaceted nature of health in cancer survivors, follow-up and survivorship care for older breast cancer survivors would ideally include comorbid disease, social support, and adverse changes in economic lifestyle.

While all breast cancer survivors may be at risk for not receiving services that may make a difference in their post-treatment quality of life, rural dwelling women may be at particular risk. Understanding the exact nature of similarities and differences in ruaral versus urban dwelling women may help ameliorate some of these risks and provide guidance as to precise nature of services that are warranted.
